# Managing the complexity of doing it all: an exploratory study on students’ experiences when trained stepwise in conducting consultations

**DOI:** 10.1186/1472-6920-14-206

**Published:** 2014-09-26

**Authors:** Leen Aper, Jan Reniers, Anselme Derese, Wemke Veldhuijzen

**Affiliations:** Faculty of Medicine and Health Sciences, Ghent University, Ghent, Belgium; SHE Academy, Faculty of Health Medicine and Life Sciences, Maastricht University, Maastricht, the Netherlands

**Keywords:** Conducting complete consultations, Didactic course principles, MeSH terms, Education, Medical, Undergraduate, Clinical competence, Physician-patient relations, Professional practice

## Abstract

**Background:**

At most medical schools the components required to conduct a consultation, medical knowledge, communication, clinical reasoning and physical examination skills, are trained separately. Afterwards, all the knowledge and skills students acquired must be integrated into complete consultations, an art that lies at the heart of the medical profession. Inevitably, students experience conducting consultations as complex and challenging. Literature emphasizes the importance of three didactic course principles: moving from partial tasks to whole task learning, diminishing supervisors’ support and gradually increasing students’ responsibility. This study explores students’ experiences of an integrated consultation course using these three didactic principles to support them in this difficult task.

**Methods:**

Six focus groups were conducted with 20 pre-clerkship and 19 clerkship students in total. Discussions were audiotaped, transcribed and analysed by Nvivo using the constant comparative strategy within a thematic analysis.

**Results:**

Conducting complete consultations motivated students in their learning process as future physician. Initially, students were very much focused on medical problem solving. Completing the whole task of a consultation obligated them to transfer their theoretical medical knowledge into applicable clinical knowledge on the spot. Furthermore, diminishing the support of a supervisor triggered students to reflect on their own actions but contrasted with their increased appreciation of critical feedback. Increasing students’ responsibility stimulated their active learning but made some students feel overloaded. These students were anxious to miss patient information or not being able to take the right decisions or to answer patients’ questions, which sometimes resulted in evasive coping techniques, such as talking faster to prevent the patient asking questions.

**Conclusion:**

The complex task of conducting complete consultations should be implemented early within medical curricula because students need time to organize their medical knowledge into applicable clinical knowledge. An integrated consultation course should comprise a step-by-step teaching strategy with a variety of supervisors’ feedback modi, adapted to students’ competence. Finally, students should be guided in formulating achievable standards to prevent them from feeling overloaded in practicing complete consultations with simulated or real patients.

**Electronic supplementary material:**

The online version of this article (doi:10.1186/1472-6920-14-206) contains supplementary material, which is available to authorized users.

## Background

In ambulatory care and family practice, meetings between doctors and patients are “consultations”. In acute hospital care, phrases like “seeing on rounds” or “conducting a complete history and physical examination” reflect a narrower focus on the integration of communication, clinical reasoning and physical examination skills [1, 2]. These doctor-patient contexts have several components in common: building rapport; identifying patients’ perspectives by exploring their ideas, concerns and expectations; obtaining information; clinical reasoning; making a diagnosis; and developing a management plan [3]. Conducting a consultation is a complex competence because physicians have to integrate parallel processes of communication, history taking, technical examination and clinical reasoning. The learning process of this complex competence is also challenging, because all the medical knowledge and skills students acquired in other parts of the curriculum before, must be adopted and integrated.

Starting before 1910, this learning process was achieved naturally. Medical education was founded wholly on apprenticeship principles whereby students learned the complex competence of consulting by “seeing one, doing one, and teaching one” [4]. Although those learners learned the different components of simple tasks at different times, they were exposed to the whole task of a consultation from the outset and did not have to integrate medical knowledge and skills acquired within other parts of the curriculum.

Later on, curricula changed because biomedical knowledge increased rapidly and therefore specific training became necessary. The Flexner reforms of 1910 added a preparatory education in biomedical science to medical students’ apprenticeship education [5]. Inspired by Michael and Edith Balint [6], general practitioners (GP) in the UK [7] started to develop communication education. First GP postgraduate curricula and then undergraduate curricula introduced communication skills training using simulated patients [8, 9]. Communication training was later sanctioned as a core component of undergraduate medical curricula by policy statements such as the UK General Medical Council’s influential first edition of “Tomorrow’s Doctors” [10]. Medical knowledge, history-taking and physical examination skills continued to be taught by practitioners alongside communication skills training by educationalists in clinical skills laboratories using simulated patients. Practitioners focused on the content of consultations; educationalist and psychologists focused on their processes. Only students continuously crossed the boundaries between those two different approaches.

Lately, it has been argued that content and process should be taught together to obtain a better transfer during clerkships. Therefore, Kurtz et al. advised to teach their communication model integrated with clinical reasoning and physical examination skills [11]. Although there is a wealth of studies evaluating methods to train communication skills, clinical reasoning skills and physical examination skills separately, there is yet little empirical information on the best methods to learn the complex task of executing complete consultations.

In educational literature there are many examples of theoretical design models that have been developed to promote the learning of complex tasks. Van Merriënboer & Sweller developed specific instructional design guidelines to manage complex learning tasks [12]. These authors state that the intrinsic load of a complex task can be managed by moving from simple to complex learning scenarios and working from low to high fidelity environments whereby the responsibility of students gradually increases. For example, asking students to perform a physical examination on a simulated patient can be defined as a simple learning scenario. Applying the complete consultation model is a complex learning scenario. Working with computer simulated patients is an example of a low fidelity environment with little responsibility for students. Later on, students’ learning can take place in more high fidelity environments whereby students conduct consultations with simulated patients or with real patients. Furthermore, Van Merriënboer & Sweller emphasize that novice learners ask different support than more experienced learners within complex tasks. For example, in the beginning students want to discuss all the important consultation elements of content in advance: What is your differential diagnosis? What do you want to explore in your physical examination? What are your findings and how would you proceed with this patient? Later on, this guidance will gradually decrease: during clerkships students might practice whole consultations with real patients and only receive feedback of their supervisor at the end.

Van Weel-Baumgarten et al. reported on their integrated consultation course in the curriculum program of Nijmegen University, Netherlands. They concluded that students highly rewarded the integrated clinical communication curriculum and due to their practice with simulated patients students felt positively prepared for practice with real patients [13]. By using questionnaires this study did not explore in depth why students appreciated this course. Which didactic principles are essential within an integrated training course to make students feel prepared for the real practice? These questions are important for medical educators evaluating and adjusting their curriculum. Therefore, this study aimed to explore in depth how students experienced the integrated consultation course and how they are influenced by this step-wise teaching of the consultation competence using the following didactic principles [12]:

- moving early from partial tasks to whole tasks
- starting with intensive support and gradually diminishing this support
- gradually raising the level of students’ responsibility by working from low to high fidelity environments.

## Methods

### Context

The research was conducted in the medical education program of Ghent University, which lasts seven years; three years to bachelors level and four years to masters level. Graduates must then complete 2–5 years of residency in their chosen specialties before they can practice independently.

**Figure 1:**
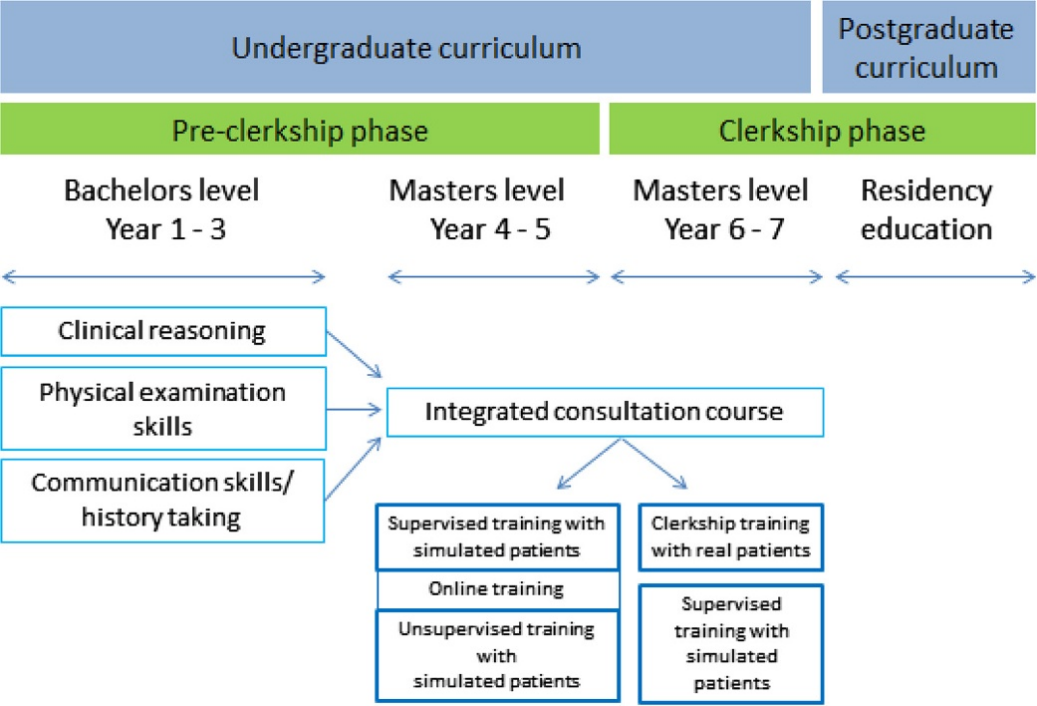
**Undergraduate curriculum design from partial tasks to whole task learning.**

In the bachelor phase students attend training sessions in communication skills, physical examination skills, and clinical reasoning separately from one another. Afterwards they learn to integrate those different consultation skills in a total of fifteen simulated patient encounters during the integrated consultation course (see Figure 1).

### Integrated consultation course

In year 4–7 the integrated consultation course uses different mutually reinforcing training formats whereby supervisors’ support gradually decreases and students’ responsibility increases (see Table 1). Students start with supervised trainings, moderated by a faculty member (GP or physician). Then they participate in an online training to prepare for the unsupervised training sessions where they only receive feedback from simulated patients and peers. Year 5 entails observational clerkships whereby students watch from the side. In year 6–7 students are on fulltime clerkships in periods of three weeks practicing on real patients under the guidance of clinical supervisors. In between these clerkships students participate once more in training sessions supervised by faculty staff.

**Table 1:** **Description of the training formats of the integrated consultation course**

Training format	Description
**1. Supervised training with simulated patients (year4 - 6)**	Students practice full consultations with simulated patients in groups of three. Each student is responsible for one part of the consultation (opening/history taking – physical examination – diagnosis, treatment and planning) whereby the student can rely on the supervising physician and peers for help. Afterwards students start with a self-reflection activity, followed by feedback from two peers and supervisor.
**2. Online training (year 5)**	An interactive web environment positions students individually in “virtual” consultations. Students are responsible for judging the consultation process and content on accuracy. The observation of small film fragments is guided by open-ended questions that prompt students about the various dimensions of consultations. Students type their answers in an input box and immediate, standardized feedback follows.
**3. Unsupervised training with simulated patients (year 5)**	Students train in pairs without supervision. Each of them conducts a full consultation with a simulated patient, while their peer observes. Feedback starts with a self-reflection activity followed by feedback of the simulated patient and peer. After the two consultations, a debriefing session take place with a physician (8–12 students) to discuss students’ questions.
**4. Clerkship training with real patients (year 6–7)**	Especially during emergency, GP training, Pediatrics and Internal Medicine clerkships students practice partial or full consultations with real patients, often in a separate room. Afterwards students debrief their clinical supervisor and observe the end of the consultation.

### Study design

This is a design based research in which we explore, qualitatively, how students experienced a theory-informed curriculum design [14–16] i.e. an integrated consultation course based on the didactic principles of Van Merriënboer et al. [12]. We chose two student groups within the learning trajectory to obtain a diversity of opinions: Year 5 students, who participated in the pre-clerkship training formats of the integrated consultation course, but who had seen clinical practice only as observers. Year 6 students, who were in their clerkships and practiced on real patients for about one year.

### Research team

The methodology of design based research is characterized by a collaboration among researchers and practitioners in real-world settings [16]. So, the research team consisted of one researcher/educationalists (LA) and three medical doctors with ample experience in medical education either in communication training as researcher (WV) or in medical skills and consultation training of students within the undergraduate curriculum (WV, JR, AD).

### Recruitment

All year 5 and 6 students were invited by email (n = 411). Eighteen students registered and additionally LA approached purposive individual students face-to-face to obtain a varied sample with regard to individual consultation skills scores and gender. Participation was voluntary and all participants gave written, informed consent.

### Data collection

We chose to explore students’ experiences in focus groups so they could build on one another’s experiences. The focus groups lasted about 90 minutes and took place during lunchtime on days when students were on campus. At the beginning, the moderator (JR) assured students that full confidentiality was guaranteed. As observer, LA kept detailed field notes during each session. During the focus groups students discussed positive and negative experiences of the time related phases within the integrated consultation course. These time related phases are a consequence of implementing the three didactic principles in our curriculum. The discussions were audiotaped and transcribed verbatim. Focus groups were scheduled until saturation was reached.

### Analysis

Transcripts were entered into NVivo Version 8 (QSR, Doncaster, Australia). An iterative process of analysis is done in line with the principles of thematic analysis [17]. All phrases related to the integrated consultation course were coded. The process of creating codes was both pre-set, created prior to data collection, and open, created while transcripts were reviewed. The pre-set codes were based on the three didactic principles and other important aspects within the integrated consultation course (see Additional file 1). The “emergent codes” stayed semantically close to participants’ own words. Next, these codes were organized in themes of interrelated codes using a constant comparative strategy in order to develop conceptualization of possible relations. All the transcripts were analyzed independently by the first two authors (LA and JR), who discussed any differences in codes after each analysis of a transcript until consensus was reached to develop a single codebook for use in the rest of the analyses. To broaden the interpretation AD and WV both coded parts of the discussions. LA, JR and WV established the relationship between the resulting themes and the didactic principles and discussed this in depth.

### Ethical approval

Ethical approval for this qualitative study was obtained from the Ethical Committee of Ghent University Hospital (registration number: B670201110504).

## Results

### Participation

Twenty pre-clerkship students participated in three focus groups (PC FG 1–3) and nineteen clerkship students participated in another three focus groups (C FG 1–3). Mean duration of the focus group sessions was 90 minutes. All sessions were characterized by animated discussions. We organized the results in relation to the descriptions of students’ experiences according the underlying didactic principles of the integrated consultation course whereby the different key concepts of each didactic principle are schematically visualized in three figures. These key concepts of each didactic principle are depicted by a specific shape (respectively rectangle, ellipse or octagon). The different shapes within the figures make it is possible to show the inter- and intra-relationships between the results. However, we are aware that distinguishing these three didactic principles is more or less artificial because they are interrelated within our curriculum. Furthermore, to enrich the results we choose explicitly to integrate specific quotes which illustrate the essence of the ‘lived’ emotions, thoughts and experiences of the students.

#### Moving from partial tasks to the whole task of a consultation

**Figure 2:**
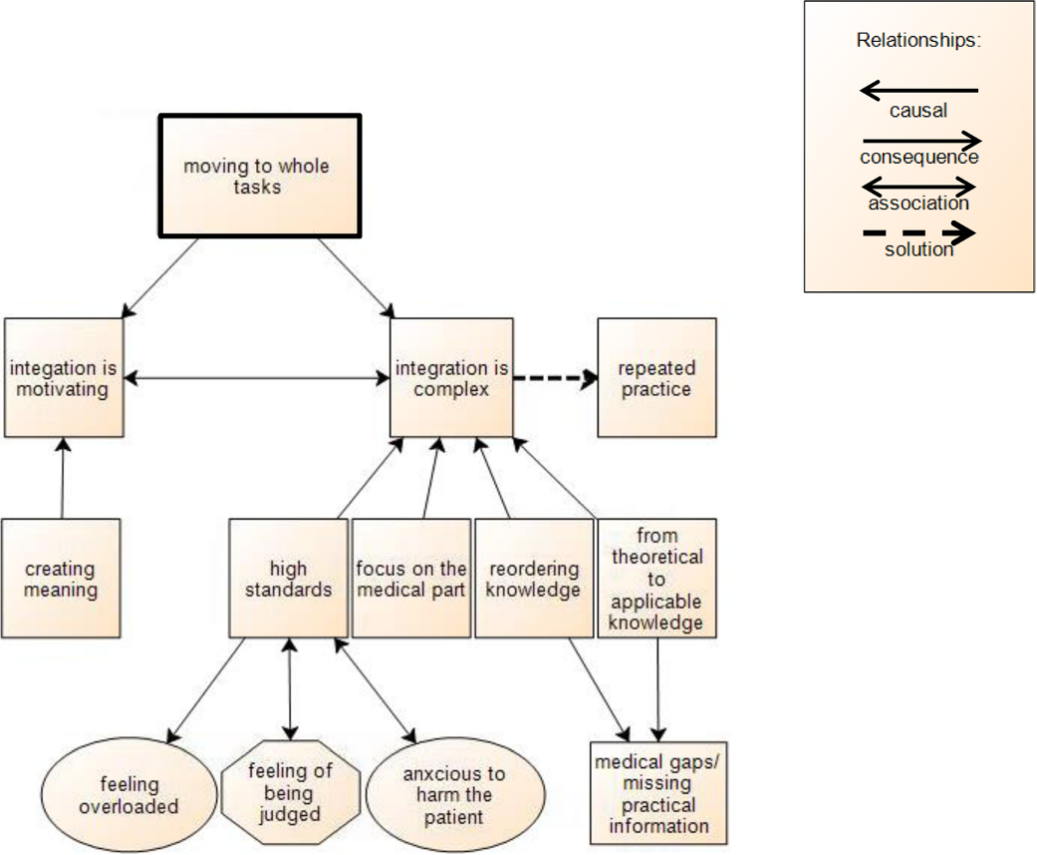
**Scheme of moving to the whole task of a consultation.**

After practicing communication skills, physical examination skills, and clinical reasoning separately in the previous years, students felt motivated to integrate their consultation skills within the structure of a complete consultation with simulated patients. As can be seen in Figure 2, this motivation is nourished by the fact that students found integrating content and process of the consultation more meaningful than doing any one of its components separately: “*The big difference is that during consultation training you have the clinical context. You have to examine the patient and make a diagnosis. In communication [training] it is less important what you say, the focus is on the way you tell it.” (PC FG2) “The integrated consultation course should start earlier, even if you do not possess the necessary theoretical and practical knowledge, then you already know: ok, that is how it works …” (PC FG2).* However, performing the whole task of a consultation was complex and frustrating, because students set high standards for themselves. Even later, they also became aware that their primary focus was more on the medical part than on the patient and that they had to transfer their theoretical knowledge into applicable clinical knowledge on the spot. Students described repeated practice of conducting consultations as a solution to get a grip on these difficulties (see Figure 2).

##### Developing the ability to manage all consultation skills at once

At first, students found it hard to integrate their clinical thinking and communication with the patient into a technically proficient interview *“… give me some time … I will get to the questions that are essential for that complaint but not in that one moment of speaking” (PC FG3)*. Mastering the medical side of the consultation, i.e. in particular clinical reasoning and physical examination, was perceived as more important and challenging by students. So during the whole task of performing a consultation students were very much focused on: *“What should I ask? … you ask several things about abdominal complaints … Do I forget something? Does the sequence of my questions make any sense?” (PC FG2).* In another example during the online training, students could address both medical content and communication process. Still they were particularly focused on their medical knowledge and clinical reasoning: *“I was preoccupied with the symptoms of the disease …” (C FG2) “I found it useful to sharpen my clinical reasoning …” (C FG1).* More advanced students found themselves paying more attention to their communication skills during the unsupervised training or during clerkships: *“ … when I felt confident about the content of the consultation I could focus more on my communication with the patient” (C FG3).* Only after repeated practice students underscored that their primary focus on the medical part seemed to regulate itself and they were able to sufficiently manage the consultation skills simultaneously. Overall, our results show students set high standards for themselves and were very much focused on performing as an expert with extensive medical knowledge rather than as a novice at the start of a long learning trajectory. For example, during some clerkships students got plenty of time to question a patient on their own but were afraid this would result in a non-focused strategy: *“… too much time leads to the risk of not being efficient in targeting the most important questions first” (C FG1)*.

##### Transferring theoretical medical knowledge into applicable clinical knowledge

Conducting complete consultations forced students to reorder their medical knowledge:*“ … in [studying] theory … you always got the diagnosis and underneath all the symptoms … too little we make the reverse link … in a consultation … the patient just tells you some symptoms … you have to check other symptoms … on the basis of a differential diagnosis …” (PC FG2)*. So, pre-clerkship students became aware that their pre-clerkship education had been too theoretical and divorced from practice to be easily applicable into real consultations. *“Often after lectures I do understand a disease but I ask my friends: what do I do when I see a real patient? What investigations do I plan? How to be sure of the diagnosis? That has not been explained clearly… the way we do it in consultation training we would remember it more easily… now we just memorize it (PC FG1).”* As a consequence, students became aware of the gaps in their medical knowledge as future physicians. Students had difficulties developing a differential diagnosis *“at this point you ask a few standard questions “fever? How long? … without having a diagnosis in your mind …” (PC FG2)* or distinguishing the most relevant complaints: *“What is important?” (C FG2).* Students looked forward to be able to prioritize: *“… during history taking I ask very broad questions … my medical thinking is not focused enough compared to my supervisors” (C FG1) “I hope the clerkships bring a kind of relief in all the flat courses we had … what is the most common?” (PC FG3).* At the end of a consultation the hands-on side of medicine needed to be addressed whereby pre-clerkship students realized they missed a lot of practical knowledge about planning and referring: *“A woman with a meniscus tear … should I first call an orthopedic surgeon or should I arrange an MRI scan? … the simulated patient asked me “Doctor, what will happen at first?” …”(PC FG1).*

#### Gradual decrease in supervisors’ feedback/support

**Figure 3:**
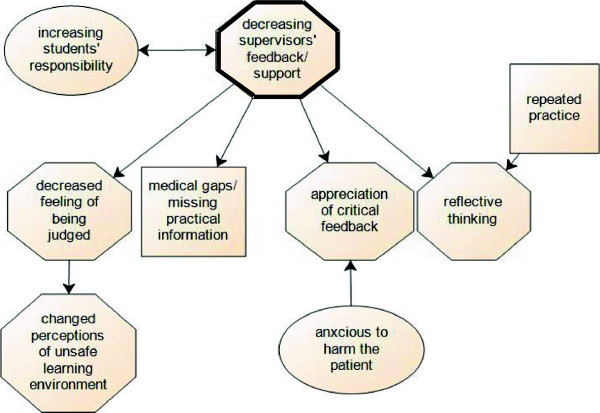
**Scheme of decreasing supervisors’ feedback/support.**

The integrated consultation course started with sessions whereby students can rely on a supervisor for feedback and support. Especially in the beginning, students found the support of a supervisor important when they had difficulties to continue the consultation or failed in their clinical reasoning: *“when we were working with simulated patients for the first time and got lost, I felt confident I could rely on the supervisor. We got feedback and could start again” (PC FG2).* “… *but it is important that there is someone next to you indicating what you are doing well or what went wrong.” (C FG2).* Contrary to our expectations, students indicated at the same time that they found it challenging to be closely supervised and judged by a physician. This resulted for students in an unsafe environment with feelings of insecurity: *“Every word we said and every logical step we took was overheard and could be wrong. That caused stress.” (*PC FG3). Decreasing the presence of a supervisor resulted in a decreased feeling of being judged, which in turn created a more safe learning environment for students (see Figure 3). Furthermore, the shift from supervisors’ support/feedback to online support and to feedback of simulated patients and peers triggered students to reflect spontaneously on their own actions and to appreciate the value of critical feedback.

##### Trigger to reflective thinking

Decreasing supervisors’ support made students aware it was up to themselves to reflect spontaneously on their actions and to be self-critical: *“I have spent 18 weeks in the same emergency department, … you have your own routine where nobody is watching. Often the consultation is not as it should be, but no one gives feedback. It is up to yourself to think about your own performance.” (C FG3)* Especially, during clerkships this reflection resulted in tapping other resources: students indicated the online training was useful at that time to practice their clinical reasoning or to correct themselves on specific physical examination tests. However, students admitted that reflecting required effort: *“If you see the doctor after your own intervention, you can evaluate very quickly what you forgot, what you did differently and where you should pay attention to next time.” But the question whether these observed actions are also correct requires an additional effort (C FG3).*

##### Appreciation of critical feedback

Pre-clerkship students felt very insecure about their consultation competence and these respondents indicated they needed a physician who highlighted the positive aspects of their performance, with only a limited amount of negative feedback. The critical feedback they sometimes received had a huge impact on the confidence of pre-clerkship students who felt vulnerable: *…I got negative feedback … and it left me with a bad feeling: “Was it that bad?”… because … you remember the negative points and positive aspects are said loosely in* between*: “ah, that was ok, that did you do well.” (PC FG1).* After repeated practice with a supervisor, pre-clerkship students became more self-confident. But the shift to online support and peer feedback included pro’s and con’s. The online support was appreciated because it helped students to be attentive to the different steps of a consultation: *“the feedback for me had a kind of alarming effect … Did I know it all? I may not forget this and that …" (PC FG3).* Concerning the peer feedback, students indicated they missed the level of medical accuracy: *“My fellow students did not know more than me … or sometimes I did not trust the reflections of my peer.” (PC FG1).* So, the moment supervisors’ support decreased, students missed their feedback, because it might have helped them to correct errors or understand nuances in the consultation structure. Later on, when clerkship students experienced only minimal support of their clinical supervisors, they started actively searching for critical feedback: *“I always asked for critical remarks during clerkships … only by receiving that [feedback] I can grow” (C FG2)*.

#### Gradual increase in students’ responsibility

**Figure 4:**
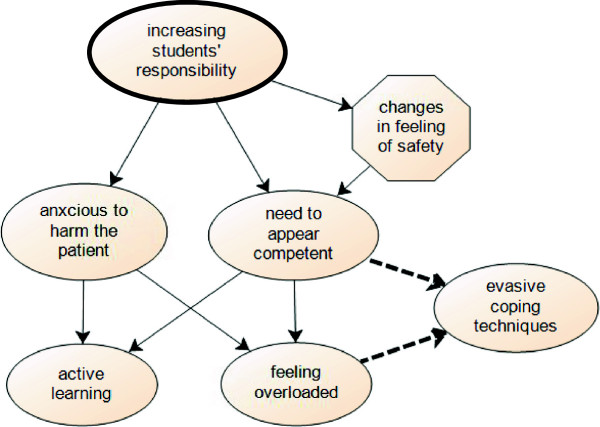
**Scheme of increasing students’ responsibility.**

The aim of the integrated consultation course is that students gradually learn to deal with responsibility by working from low to high fidelity environments. During the supervised sessions students appreciated the time-out discussion with the supervisor concerning the medical content *“I felt prepared to perform that part of the consultation with the simulated patient”*. During the online training students indicated their sense of responsibility was less addressed because there was no real interaction with the patient. During the unsupervised training sessions students became responsible to integrate content and process in a consultation role play with a simulated patient. Students experienced these sessions as very close to reality, creating a large feeling of responsibility. Later on, this feeling of responsibility made students anxious not to harm the patient and made them feel the need to appear competent (see Figure 4). So, despite the preparation within the simulated setting, the transition towards having responsibility for real patients remained difficult. In year six students were immersed into the real world: *“It was a big step … I was thrown immediately into the emergency room … and suddenly I had to start doing everything myself. … you are stunned …”(C FG1)*. As Figure 4 visualizes, the anxiousness to harm the patient and the need to appear competent had the advantage of stimulating active learning but unfortunately, some students felt overloaded even within this gradual teaching approach.

##### Stimulating active learning

Throughout the medical curriculum students were accustomed to follow theoretical lessons and passively observing physicians. Increasing students’ responsibility made pre-clerkship students understand that being able to reproduce their theoretical knowledge was not enough, they needed to apply this knowledge: *“… how do I make a correct diagnosis? … until now I was just memorizing …”(PC FG2)*. During the online training students evaluated their medical knowledge “… *I could test myself … “Would I have asked those questions too?. (PC FG1)”* But the responsibility towards real patients during clerkship training activated students’ thinking process the most: “… *I started to make little lists of what I should ask …”(C FG1)*.

##### Feeling overloaded

Apparently, even with our stepwise teaching approach of increasing students’ responsibility, some respondents still felt overloaded. Students wanted to appear competent but were anxious to miss important information, not to be able to answer patients’ questions or not to take the right decisions: *“I find the unsupervised training very valuable, … but I experienced also a feeling of insecurity, it is up to me to make the decisions” (PC FG1). “It is with extremes, … one clerkship I got responsibility for the therapy … It was my very first clerkship, I was not ready for it yet …” (C FG3).* Some students who had difficulties with this responsibility when they acted autonomously were adopting evasive coping techniques as a solution, even within the simulated setting: *“I started talking very fast with my patient hoping she wouldn’t ask me any further questions” (PC FG1).*

## Discussion

### Main results

This study explores three didactic principles based on the instructional design guidelines of Van Merriënboer & Sweller [12] (i.e. moving early from partial tasks to whole tasks, gradually diminishing the supervisors’ support, gradually raising the level of responsibility of students by working from low to high fidelity environments) within an integrated consultation course where students learn the process of conducting consultations. Implementing the whole task of consulting with real patients within medical curricula makes students aware that they have to reorganize their medical knowledge and transfer their theoretical medical knowledge into applicable clinical knowledge. Furthermore, it stimulates students’ active learning and triggers their reflective thinking. Within this stepwise teaching approach managing the consultation skills simultaneously remains difficult, because students set high standards for themselves with the risk of feeling overloaded. Initially, students have their primary focus on the medical part of the consultation (e.g. having extensive medical knowledge, being able to recognize the most important symptoms, focusing their history taking sufficiently) with high expectations about time efficiency. Furthermore, students are vulnerable and afraid of receiving negative feedback. However, after repeated practice they are able to perform the basics of the consultation process and want to refine their competence by explicitly asking for critical feedback.

### Comparison to the literature

Our results show that dealing with the complexity of a complete consultation forces students to transfer their theoretical medical knowledge into applicable clinical knowledge. This is in line with Prince et al. that students do not seem to have the appropriate knowledge readily available [18]. Our respondents agree that the clinical practice called for a different type of clinical knowledge as compared to what they acquired during pre-clinical training. Furthermore, the focus group discussions tell us that students experience their thinking process is not quick enough to pose the right questions in relation to a particular complaint. Mandin et al. stress the fact that the inability to recall information stored in memory is due to lack of cognitive organization and understanding [19], which underpins students’ perceived need to reorganize information. Therefore, Prince et al. and Bombeke et al. suggest that the pre-clerkship curriculum should organize structured rehearsal of integrated skills and set up reflective conversations in the clinical phase [18, 20].

Earlier studies state that working with simulated patients eases the transition to real patients contact within medical curricula [9, 21]. Nevertheless, our study shows that conducting consultations with real patients remains a difficult transition, that entails a huge jump in responsibility for students, but also activates their thinking process the most. We agree with Bokken et al. and Spencer et al. who state that real patients make a more profound impression on students [22] and therefore promote the relevance of students’ learning [23].

Our results show that students set high standards for themselves when they are exposed to the experience of performing a consultation for the first time. This can be explained by the fact that learning to conduct complete consultations is a moment of transfer whereby students have to deal with new expectations and new responsibilities [24]. This is implicated in the research of Verdonk et al. who indicate that medical students perceive the medical culture as hierarchical and competitive where they have to present themselves continuously as professional and self-confident [25].

Finally, our findings demonstrate the dilemma that exists between diminishing the supervisors’ support when the consultation competence of students grows, and the need of students for critical feedback when they are able to perform the basics of consulting in a context of increasing responsibility. Similar to our results, Bok et al. have found that during clinical clerkships, students actively seek feedback when they have responsibilities in patient care [26]. Other studies have shown that the feedback of a supervisor, who is standing higher in clinical hierarchy, is perceived by medical students as better compared to feedback from peers or paramedical staff [27, 28].

### Limitations

Despite the interesting findings of this study some of its limitations need to be addressed. In the present study only a specific student sample was included from one university in Belgium. However, using well defined didactic principles within our research question make our findings relevant for other universities who might wish to integrate these didactic principles within their own training formats. The concept of ‘early’ moving from partial to whole tasks learning can be interpreted differently within every medical curriculum. In our curriculum ‘early’ means starting two years before clerkships (year 4), which is a bit late in the curriculum compared to other universities. Maastricht University for example, already starts with whole task learning in year 1 [29]. Further research can involve other participants like educational staff and clinical supervisors as their complementary experiences might enrich our understanding of the gains and pitfalls as students learn to integrate the complex task of performing a consultation.

## Conclusion

Moving early to whole task learning of consultations within medical curricula with decreasing supervisors’ support and increasing students’ responsibility had several advantages. Students’ initial primary focus on the medical part regulated itself and students were motivated to pay attention to their communication skills. Furthermore, students became aware they should transfer their theoretical knowledge into applicable clinical knowledge. They were stimulated in their active learning and triggered in their reflective thinking. Paradoxically, starting with intensive supervisors’ support and diminishing this support gradually did not match with students’ needs for critical feedback. A variety of supervisors’ feedback modi, adapted to students’ consultation competence, should be provided. But, even within this step by step teaching approach the transition to being responsible for real patients remained difficult and overwhelming for some students. Supervisors should help students in formulating achievable standards throughout their learning trajectory to prevent them from feeling overloaded and adopting evasive coping techniques.

### Implications

– This study emphasizes the importance of incorporating the practice of complete consultations early within medical curricula, in order to give students the time to reorganize their knowledge before they are immersed into the real world.
– Instead of decreasing supervision during the integrated consultation course, it is important to explore how supervisors’ feedback can remain present and adaptive to the competence of the students during their learning trajectory. Video recording of students’ consultations can offer a solution whereby students decide on which part of the consultation they want feedback from a supervisor.
– The fact that conducting consultations with real patients activates students’ thinking process the most, underlines the importance of sufficient pre-clinical training opportunities with real patients and real decisions in a safe environment [30]. However, a huge effort is expected from supervisors to achieve specific learning goals in these real patients contacts. Ideally, a tailor-made student approach with different supervisors’ feedback modi is needed, but logistically difficult to achieve.
– This study shows that students seem to have difficulty to realize they are at the start of a long learning trajectory in consulting whereby they gradually transfer theoretical knowledge into applicable knowledge. Medical students may benefit from Jacobson who describes in essence what the beginning of a learning trajectory in consulting is about: “within the utility of the medical student interview … the biggest gift is time … these early experiences will shape the clinicians we will become. The skills of patience and empathy are inherent, but … need practice … a medical student has ample opportunity to practice these important skills …” [31].

## Electronic supplementary material

Additional file 1: **List of “pre-set” codes.** (DOCX 13 KB)

## References

[1] Menahem S (1987). Interviewing and examination skills in paediatric medicine: videotape analysis of student and consultant performance. J R Soc Med.

[2] Sutter C, Reid T (2012). How do we talk to the children? Child life consultation to support the children of seriously ill adult inpatients. J Palliat Med.

[3] Veening EP, Gans ROB, Kuks JBM (2009). Medische Consultvoering: Hoofdlijnen en Achtergronden. [Conducting medical consultations: outlines and backgrounds].

[4] Lave J, Wenger E (1991). Situated Learning: Legitimate Peripheral Participation.

[5] Cooke M, Irby DM, O’Brien BC (2010). Educating Physicians: A Call for Reform of Medical School and Residency. Carnegie Foundation for the Advancement of Teaching.

[6] Balint M (1963). The Doctor, his Patient, and the Illness.

[7] Pendleton D, Schofield T, Tate P, Havelock P (1984). The Consultation: an Approach to Learning and Teaching.

[8] Van Dalen J, Zuidweg J, Collet J (1989). The curriculum of communication skills teaching at Maastricht Medical School. Med Educ.

[9] Bokken L, Rethans JJ, Scherpbier AJ, van der Vleuten CP (2008). Strenghts and weaknesses of simulated and real patients in the teaching of skills to medical students: a review. Simul Healthc.

[10] General Medical Council (1993). Tomorrow’s Doctors.

[11] Kurtz S, Silverman J, Benson J, Draper J (2003). Marrying content and process in clinical method teaching: enhancing the Calgary-Cambridge guides. Acad Med.

[12] Van Merriënboer J, Sweller J (2010). Cognitive load theory in health professional education: design principles and strategies. Med Educ.

[13] van Weel-Baumgarten E, Bolhuis S, Rosenbaum M, Silverman J (2013). Bridging the Gap: how is integrating communication skills with medical content throughout the curriculum valued by students?. Patient Educ Couns.

[14] Edelson DC (2002). Design research: what we learn when we engage in design. J Learn Sci.

[15] Cobb P, Confrey J, DiSessa A, Lehrer R, Schauble L (2003). Design experiments in educational research. Educ Res.

[16] Wang F, Hannafin MJ (2005). Design-based research and technology-enhanced learning environments. ETR&D-Educ Tech Res.

[17] Braun V, Clarke V (2006). Using thematic analysis in psychology. Qual Res Psychol.

[18] Prince KJ, Boshuizen HP, van der Vleuten CP, Scherpbier AJ (2005). Students’ opinions about their prepartion for clinical practice. Med Educ.

[19] Mandin H, Jones A, Woloschuk W, Harasym P (1997). Helping students learn to think like experts when solving clinical problems. Acad Med.

[20] Bombeke K, Symons L, Vermeire E, Debaene L, Schol S, De Winter B, Van Royen P (2012). Patient-centredness from education to practice: the ‘lived’ impact of communication skills training. Med Teach.

[21] Bokken L, Rethans JJ, van Heurn L, Duvivier R, Scherpbier A, van der Vleuten C (2009). Students’ views on the use of real patients and simulated patients in undergraduate medical education. Acad Med.

[22] Bokken L, Rethans JJ, Jöbsis Q, Duvivier R, Scherpbier A, van der Vleuten C (2010). Instructiveness of real patients and simulated patients in undergraduate medical education: a randomized experiment. Acad Med.

[23] Spencer J, Blackmore D, Heard S, McCrorie P, McHaffie D, Scherpbier A, Gupta TS, Singh K, Southgate L (2000). Patient-oriented learning: a review of the role of the patient in the education of medical students. Med Educ.

[24] Teunissen PW, Westerman M (2011). Opportunity or threat: the ambiguity of the consequences of transitions in medical education. Med Educ.

[25] Verdonk P, Räntzsch V, de Vries R, Houkes I (2014). Show what you know and deal with stress yourself: a qualitative interview study of medical interns’ perceptions of stress and gender. BMC Med Educ.

[26] Bok H, Teunissen P, Spruijt A, Fokkema J, van Beukelen P, Jaarsma D, van der Vleuten C (2013). Clarifying students’ feedback-seeking behaviour in clinical clerkships. Med Educ.

[27] Ilgen D, Fisher C, Taylor M (1979). Consequences of individual feedback on behavior in organizations. J Appl Psychol.

[28] Van Hell EA, Kuks JB, Raat AN, Van Lohuizen MT, Cohen-Schotanus J (2009). Instructiveness of feedback during clerkships: influence of supervisor, observation and student initiative. Med Teach.

[29] Van Dalen J, Bartholomeus P, Kerkhofs E, Lulofs R, Van Thiel J, Rethans J-J, Scherpbier AJJA, Van der Vleuten CPM (2001). Teaching and assessing communication skills in Maastricht: the first twenty years. Med Teach.

[30] Widyandana D, Majoor G, Scherpbier A (2010). Transfer of medical students’ clinical skills learned in a clinical laboratory to the care of real patients in the clinical setting: the challenges and suggestions of students in a developing country. Educ Health.

[31] Jacobson D (2014). Through the eyes of a medical student. Perspect Med Educ.

[CR32] The pre-publication history for this paper can be accessed here:http://www.biomedcentral.com/1472-6920/14/206/prepub

