# Supplementary material for: Managing the complexity of doing it all: an exploratory study on students’ experiences when trained stepwise in conducting consultations
Source: BMC Med Educ. 2014 Sep 26;14:206. doi: 10.1186/1472-6920-14-206 (PMC4181426; doi:10.1186/1472-6920-14-206)
Supplement: Supplementary file 1 — Additional file 1: List of “pre-set” codes. (DOCX 13 KB) [file 12909_2014_1027_MOESM1_ESM.docx]

Additional file 1: List of “pre-set” codes

- separate skills versus integration of skills
- practicing consultations with simulated patients versus real patients
- presence of supervisor versus absence of supervisor
- observation of peers versus online film fragments
- feedback of supervisor versus feedback of peers
- self-confidence in consulting
- impact of responsibility
- importance of feedback versus lack of feedback
- self-reflection in consulting
